# A crop sequence dataset of the German federal state of North Rhine-Westphalia from 2019–2024

**DOI:** 10.1016/j.dib.2025.111617

**Published:** 2025-05-07

**Authors:** Christoph Pahmeyer, Till Kuhn, Hugo Storm

**Affiliations:** a Thünen Institute of Farm Economics, Bundesallee 63, 38116 Braunschweig, Germany; b Institute for Food and Resource Economics, Data Science in Agricultural Economics Group, Meckenheimer Allee 174, 53115 Bonn, Germany

**Keywords:** Crop rotation, IACS data, LPIS data, Agricultural geodata, Spatial data processing, Agricultural monitoring

## Abstract

This paper presents a comprehensive dataset of crop sequences for agricultural fields in the German federal state of North Rhine-Westphalia, covering the period from 2019 to 2024. The dataset encompasses approximately 1,444,096 hectares of agricultural land and 740,731 field parcels, provided in vector format (GeoParquet, WGS84). While annual crop type data is commonly available through agricultural subsidy records, tracking crop sequences over time is challenging due to changing field boundaries between years. This dataset was created using a novel spatial joining approach that tracks crop sequences at the field level by determining the largest area overlap between years, achieving a 97 % matching rate compared to the 24 % reported in previous field-identifier-based approaches. The underlying crop type data was sourced from the OpenGeodata portal of North Rhine-Westphalia. Besides the specific dataset we also provide an open-source Python package, available on GitHub. This dataset enables detailed analyses of crop sequences, assessment of agricultural practices, and evaluation of environmental impacts while maintaining the spatial integrity of management units, making it particularly suitable for agricultural modeling, ex-post policy assessment, and farm management studies. With access to IACS data becoming increasingly available in EU member states, this methodology can be applied beyond the case study region.

Specifications Table

**Table:** 

Subject	Earth & Environmental Sciences
Specific subject area	Crop sequence analysis, Agricultural land use
Type of data	Vector geodata (GeoParquet)
Data collection	Data processing of official agricultural land use data from OpenGeodata NRW using a spatial joining algorithm
Data source location	North Rhine-Westphalia, Germany
Data accessibility	Repository name: https://github.com/UniBonn-DataScience-in-AgEcon-Group/Crop_Sequences_Data_Article Data identification number: 10.5281/zenodo.15011155 Direct URL to data: https://zenodo.org/records/15011155/files/Crop_Sequences_NRW_2019_2024.parquet?download=1
Related research article	None

## 1 Value of the Data

• This dataset provides continuous crop sequences for approximately 741,000 agricultural fields in North Rhine-Westphalia over a six-year period (2019–2024), enabling detailed analyses of agricultural land use patterns, and serves as a valuable resource for studying land use changes and their environmental impacts.
• The vector-based approach preserves field boundaries and spatial relationships, offering advantages over raster-based approaches for analyzing crop sequences and supporting environmental monitoring.
• The dataset can be used to assess agricultural sustainability, evaluate compliance with environmental policies, study the diversity of crop sequences at various scales, and inform socio-economic analyses of agricultural practices.
• Researchers can use this dataset to study relationships between crop sequences and environmental factors, such as soil quality, water quality, biodiversity, and other ecosystem services.
• The accompanying open-source Python script enables researchers to replicate the methodology for other regions where annual crop type data is available, promoting standardized approaches to crop sequence analysis and facilitating broader applications across disciplines.

## 2 Background

The compilation of this dataset is motivated by the need for accurate, field-level crop sequence information to support agricultural policy evaluation and farm management analysis. While annual crop type data is readily available through agricultural subsidy records, existing methods for tracking crop sequences over time have strong limitations. Previous work in the field mainly relies on two methods: Field-identifier and raster-based approaches for crop sequence matching. Field-identifier-based approaches typically achieve only low matching rates, while raster-based methods lose the spatial integrity of management units. However, many analytical applications - including crop sequence analysis, economic modeling of policy impacts, and assessment of soil organic carbon dynamics - require reliable information about predominant crop sequences at the field level. This practical need drives the development of a spatial joining methodology that maintains field boundaries while achieving high temporal matching rates.

## 3 Data Description

The dataset consists of a single GeoParquet file (approximately 354 MB) containing crop sequence information for agricultural fields in North Rhine-Westphalia, Germany, covering the period from 2019 to 2024. The dataset includes approximately 741,000 field parcels represented as polygon geometries in the WGS84 coordinate reference system. The field geometries correspond to the field boundaries as recorded in 2024, the most recent year of the dataset.

The underlying data originates from the Integrated Administration and Control System (IACS), which is the European Union's primary system for managing and controlling payments to farmers under the Common Agricultural Policy (CAP). As part of the IACS, the land parcel identification system (LPIS) provides the geometries and location of agricultural fields. IACS data includes not only detailed spatial information about agricultural parcels but also about their use, as farmers must annually declare their cultivated crops for subsidy payments. This system results in highly accurate and regularly updated agricultural land use data across the European Union. Similar datasets are available for most member states through various national and regional geodata portals, as documented in the EuroCrops dataset [1].

Each record in the dataset represents a single agricultural field and includes a unique field identifier along with the crop types cultivated on that field for each year from 2019 to 2024. Crop types are coded according to the standardized German crop classification system used in the IACS. This three-digit coding system hierarchically represents crop information: the first digit indicates the main crop group, the second digit specifies the crop group, and the third digit identifies the specific crop variety. For example, in the code ‘131’, ‘1’ represents the wheat group, ‘3’ indicates barley, and ‘1’ specifies winter barley. The complete classification system and corresponding codes can be accessed through the official website of the Chamber of Agriculture of North Rhine-Westphalia [[2], [3], [4], [5], [6], [7]].

The dataset's quality is assured by its origin in IACS data, which is used for agricultural subsidy payments and thus subject to regular verification. While the dataset may not include fields from farms that do not receive subsidies, it encompasses the vast majority of agricultural land in North Rhine-Westphalia. The high quality of the data is further ensured through various administrative controls, including on-site inspections and remote sensing-based verification, which are regularly conducted as part of the CAP control system.

## 4 Materials and Methods

**Fig. 1:**
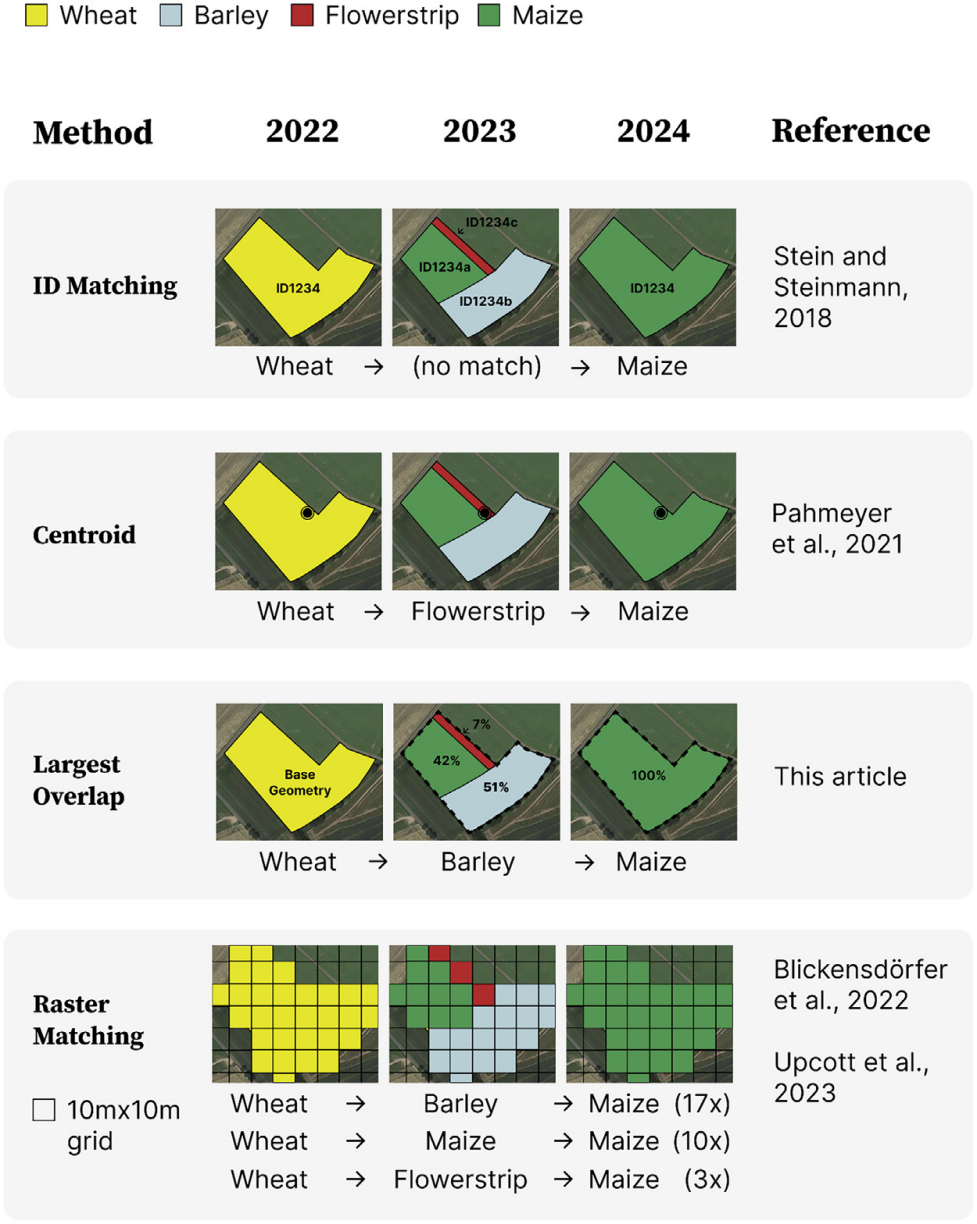
Overview of the different crop sequence identification methodologies found in the literature. Source: Own illustration.

Different approaches have been developed to determine crop sequences from annual agricultural land use data. These methods vary in their computational complexity, information preservation, and practical relevance for agricultural management. Fig. 1 illustrates four main approaches using an example field that undergoes splitting and merging between 2022 and 2024, demonstrating how each method handles such boundary changes differently.

The most straightforward approach is based on matching field identifiers across years [8], shown in Fig. 1 as the “ID matching” method. This method relies on administrative field IDs remaining constant over time. The method is computationally efficient and does not require GIS knowledge (e.g. can be performed in Spreadsheets). However, its major limitation is the substantial loss of information when field boundaries or identifiers change. Stein and Steinmann [8] reported that only 24 % of parcels could be matched over their study period (2005–2011) using this method, highlighting the need for more robust approaches. Compared to our proposed largest overlap method, ID matching significantly compromises accuracy and completeness in determining crop sequences, as it fails to account for changes in field boundaries over time. The largest overlap method overcomes this limitation by considering spatial relationships and the predominant crop type across consecutive years, thus maintaining both data consistency and practical relevance.

A second approach uses field centroids to track crop sequences (Fig. 1, “Centroid” method) [9]. In this method, a centroid is calculated from the field geometry in the initial year, and crops are determined by the land use at this point in subsequent years. While this method preserves more information than the field ID approach and remains computationally efficient, it can produce misleading results when the centroid falls in a minor portion of a split field or when slight boundary shifts cause the point to fall outside field boundaries.

The third method, which we implement in this study and illustrate in Fig. 1 under “Largest Overlap”, determines crop sequences through the largest area overlap between consecutive years. This approach identifies the predominant crop sequence by considering the spatial relationship between field geometries across years. When a field is split or merged, the crop type covering the largest overlapping area is used to construct the sequence. While computationally more demanding than the previous methods, this approach better reflects the primary crop sequence practiced on the field and maintains the spatial integrity of agricultural management units.

The fourth approach (Fig. 1, “Raster” method) converts vector field boundaries to high-resolution raster data, typically at 10-meter resolution, enabling the identification of multiple crop sequences within original field boundaries [10,11]. This method provides the most detailed view of crop sequence patterns and can capture marginal elements such as buffer strips. However, it requires significant computational resources for the vector-to-raster conversion and loses the spatial context of management units. Moreover, the resulting multitude of sequences may not align with farmers' planned rotations, which are typically implemented at the field level. As this approach does not maintain the plot boundaries it is also ill-suited for all analyses that aim to study crop rotation choices where the plot (or the field) is the relevant decision-making unit.

Each method represents a trade-off between computational efficiency, information preservation, and practical relevance. While raster-based approaches offer high spatial detail, they may not best serve applications focused on farm-level decision making or agricultural policy assessment. The largest overlap method we present strikes a balance between these factors, maintaining field-level management units while capturing the predominant crop sequences actually implemented by farmers.

This approach offers significant practical implications for agricultural management and policy development. Farmers can use the results to refine their crop rotation plans, ensuring optimal use of field-level management units. By accurately identifying predominant crop sequences, this method supports knowledge of current farming practices and helps identify steps towards more sustainable farming and efficient resource allocation.

Policymakers, on the other hand, can apply these findings to design agricultural policies that address field-level variability in land use and crop rotation. This method can aid in identifying trends in agricultural land management, enabling more targeted support programs or regulations regarding crop diversity, as, for instance, foreseen in the Conditionality or Eco-Schemes of the EU Common Agricultural Policy.

In the next subsection, we provide a detailed description of the largest overlap methodology, including the algorithmic implementation and computational considerations. The subsequent subsection then describes the underlying agricultural land use data for North Rhine-Westphalia and its processing into the final crop sequence dataset.

### 4.1 Largest overlap methodology

The largest overlap methodology is implemented in Python using the GeoPandas library for spatial operations. The algorithm processes agricultural field data sequentially, starting from the most recent year and working backward through the temporal sequence. For each field in year t, it determines the corresponding field and crop type in year t-1 based on the largest overlapping area between field geometries. Fig. 1, the “Largest Overlap” method, illustrates this process for a field that undergoes splitting and merging between 2022 and 2024.

Formally, for each field $F_{i,t}$ in year t, the algorithm identifies all fields $F_{j,t-1}$ in year t-1 that spatially intersect with $F_{i,t}$. The intersection area $A_{i,j}$ between $F_{i,t}$ and each $F_{j,t-1}$ is calculated as:

$$A_{i,j}=area\left(F_{i,t}\cap F_{j,t-1}\right)$$

The field $F_{j,t-1}$ with the maximum intersection area is then selected to determine the crop type for year t-1 in the sequence:

$F_{\max,t-1}=\arg\max_{j}\left(A_{i,j}\right)$ for all j where $A_{i,j}>1m^{2}$

A minimum threshold of 1m² is applied to exclude negligible overlaps that may result from minor geometric inconsistencies or digitization errors. The crop type of $F_{\max,t-1}$ is then assigned to the sequence for year t-1.

The vector-based implementation is computationally efficient, with the dataset processing taking approximately 12 min on a 2021 MacBook Pro M1 - equivalent to 2.4 min per joined year. While spatial joining is inherently more computationally intensive than raster-based approaches, it preserves detailed vector geometry and avoids information loss during raster-vector format conversion. Comparisons with alternative methods, such as the raster-based approach of Upcott et al [10] or the field-grouping approach of Stein and Steinmann [8], are challenging due to the absence of reported performance metrics in their studies. However, the lack of spatial joining in Stein and Steinmann’s method suggests a higher speed at the cost of detailed geometric relationships, while the raster-based approach by Upcott et al [10] is likely faster, as raster operations are generally more computationally efficient than vector-based joins.

The technical implementation consists of the following steps:

1. Data Import and Preparation:

plots_current = gpd.read_file(current_year_file)

plots_current.set_crs(epsg=25832, inplace=True)

2. Spatial Join Operation:

plots_current = plots_current.sjoin(

  crop_types[year],

  how='left',

  predicate='intersects'

)

3. Intersection Area Calculation:

plots_current["intersection"] = plots_current.apply(

  lambda row: row['geometry'].intersection(

   row['geometry_right']).area if row['geometry_right'] else 0,

  axis=1

)

4. Selection of Maximum Overlap:

plots_current = plots_current\

  sort_values(by='intersection')\

  groupby('ID_left')\

  last()\

  reset_index()

For quality assessment and transparency, the algorithm can store all intersecting fields and their overlap areas in a separate column using the following format:

field_id_overlap_area_crop_type::field_id_overlap_area_crop_type

For example:

12345_10000_115::12346_5000_115

indicates that the current field intersects with two historical fields: field 12,345 with an overlap of 10,000 m² growing crop type 115, and field 12,346 with an overlap of 5000 m² also growing crop type 115.

The script can be executed from the command line using three main arguments:

python join-plots.py –cur <current_year_file> –hist

<historical_files_folder> –out <result_file>

where `current_year_file` is the path to the most recent year's field data, `historical_files_folder` contains the field data for previous years, and `result_file` specifies the output location. The script accepts common geospatial vector formats including Shapefile, GeoPackage, and GeoJSON, and can output to various formats including GeoParquet for efficient storage of large datasets.

For example, to create crop sequences for another German federal state, one could use:

python join-plots.py –cur ./data/2024.parquet –hist ./historical_data –out ./Crop_Sequences_2019-2024.parquet

assuming the input data follows the same structure as the IACS data, with field geometries and crop codes stored in yearly files. Additional arguments `–key` and `–id` can be specified if the column names for crop types and field identifiers differ from the default values.

### 4.2 IACS source data and processing

The crop sequence dataset is derived from official IACS data published through the OpenGeodata portal of North Rhine-Westphalia. The portal provides regularly updated agricultural land use data as part of its environmental and climate data collection. Two primary source datasets were used: the current year's field parcels (“Beantragte und als förderfähig festgestellte Teilschläge”) [12] and the historical parcel data (“Hist. beantragte und als förderfähig festgestellte Landschaftselemente”) [13].

The source data contains comprehensive attribute information including unique field identifiers (ID, INSPIRE_ID), physical block references (FLIK), area measurements (AREA_HA), crop codes (CODE) with corresponding text descriptions (CODE_TXT), and broader use categories (USE_CODE, USE_TXT). The crop coding system differentiates between major agricultural categories including arable fodder, industrial crops, permanent grassland, permanent cultures, protein crops, energy crops, vegetables, cereals, root crops, herbs, and oilseeds.

To facilitate efficient processing, the source data was converted from Shapefile format to GeoParquet using GDAL/OGR tools. A preprocessing script was developed to handle the specific structure of the NRW data, particularly to separate the historical dataset into yearly files as required by the joining algorithm. The preprocessing workflow is automated through a shell script that downloads the current data, converts formats, and splits the historical dataset by year.

The resulting dataset provides a full 6-year crop sequence for 97 % of the geometries from the year 2024. Unmatched fields primarily result from gaps in the source data, which can occur when fields are not eligible for CAP subsidies or have been converted to non-agricultural use. The high matching rate is particularly noteworthy given the challenges of field boundary changes discussed in previous studies.

Table 3 presents a comparison of crop shares between the original datasets for each year and the crop shares resulting from the crop sequences of the largest overlap method. On average, the resulting crop shares deviate by 1,7 % from the original, with a maximum deviation of 7 % for a select few cases (e.g. arable grass in 2019 and 2020).

**Table 3:** Similarity of crop shares in the original data (AREA_HA) and the results file from the largest overlap method (AREA_HA_Result) for the 10 most cultivated crops in the years 2019–2024.

Year	CODE	NAME	AREA_HA	AREA_HA_Result	AREA_SIMILARITY_PERC
2024	459	Permanent grassland	409.412,43	398.442,54	97,3
2024	411	Silage maize	203.490,72	201.973,90	99,3
2024	115	Winter wheat	188.193,30	187.171,07	99,5
2024	131	Winter barley	136.337,65	135.703,89	99,5
2024	171	Grain maize	84.720,98	84.386,80	99,6
2024	603	Sugar beets	58.032,10	57.684,76	99,4
2024	311	Winter rapeseed	51.672,96	51.385,59	99,4
2024	602	Potatoes	45.611,29	45.261,14	99,2
2024	424	Arable grass	35.313,27	34.256,63	97,0
2024	156	Winter triticale	35.144,62	35.018,14	99,6
2023	459	Permanent grassland	411.028,58	397.892,14	96,8
2023	115	Winter wheat	237.375,11	235.751,88	99,3
2023	411	Silage maize	194.549,74	191.670,33	98,5
2023	131	Winter barley	138.362,69	137.693,91	99,5
2023	171	Grain maize	71.138,22	70.087,85	98,5
2023	311	Winter rapeseed	60.998,58	60.849,12	99,8
2023	603	Sugar beets	53.165,24	53.211,79	100,1
2023	156	Winter triticale	51.290,87	50.956,55	99,3
2023	121	Winter rye	43.357,65	42.878,68	98,9
2023	602	Potatoes	41.330,06	40.941,45	99,1
2022	459	Permanent grassland	413.902,36	397.256,36	96,0
2022	115	Winter wheat	231.640,77	229.744,35	99,2
2022	411	Silage maize	200.608,00	195.784,96	97,6
2022	131	Winter barley	132.349,86	131.338,17	99,2
2022	171	Grain maize	86.486,41	84.967,16	98,2
2022	156	Winter triticale	55.767,54	54.882,05	98,4
2022	603	Sugar beets	53.434,73	53.379,09	99,9
2022	311	Winter rapeseed	48.348,53	48.211,66	99,7
2022	602	Potatoes	39.311,29	38.545,36	98,1
2022	121	Winter rye	36.817,25	36.262,22	98,5
2021	459	Permanent grassland	416.189,96	396.834,77	95,3
2021	115	Winter wheat	222.761,23	220.505,55	99,0
2021	411	Silage maize	215.589,92	209.454,67	97,2
2021	131	Winter barley	139.111,19	137.661,96	99,0
2021	171	Grain maize	79.490,20	78.251,36	98,4
2021	156	Winter triticale	56.922,14	55.846,92	98,1
2021	603	Sugar beets	53.258,42	53.043,48	99,6
2021	311	Winter rapeseed	41.824,56	41.611,52	99,5
2021	121	Winter rye	39.695,80	38.913,38	98,0
2021	602	Potatoes	37.316,65	36.706,51	98,4
2020	459	Permanent grassland	417.049,44	396.313,95	95,0
2020	115	Winter wheat	223.937,56	221.267,60	98,8
2020	411	Silage maize	217.900,87	211.652,34	97,1
2020	131	Winter barley	147.727,62	145.921,47	98,8
2020	171	Grain maize	81.261,21	79.873,38	98,3
2020	156	Winter triticale	59.323,83	58.216,07	98,1
2020	603	Sugar beets	50.984,19	50.886,63	99,8
2020	311	Winter rapeseed	40.440,91	40.278,68	99,6
2020	602	Potatoes	38.457,69	37.788,83	98,3
2020	424	Arable grass	35.810,36	33.319,48	93,0
2019	459	Permanent grassland	417.349,68	395.775,35	94,8
2019	115	Winter wheat	244.827,71	241.317,31	98,6
2019	411	Silage maize	210.235,09	203.870,39	97,0
2019	131	Winter barley	147.256,56	145.095,98	98,5
2019	171	Grain maize	83.252,14	81.721,50	98,2
2019	156	Winter triticale	62.359,26	61.146,04	98,1
2019	603	Sugar beets	57.898,05	57.755,64	99,8
2019	311	Winter rapeseed	40.535,27	40.044,64	98,8
2019	602	Potatoes	37.539,13	36.716,34	97,8
2019	424	Arable grass	34.934,97	32.376,77	92,7

## Limitations

While the largest overlap methodology offers a robust means of constructing crop sequences, several limitations should be acknowledged. First, the assumption that the field with the largest overlapping area represents the primary crop sequence may not always hold true, especially in cases of frequent field splitting, merging, or significant boundary changes. This can lead to misrepresentation of actual agricultural practices, particularly in diverse cropping systems or where intercropping and polyculture occur.

Second, focusing on field-level management units may overlook intra-field variations such as buffer strips or sub-field cropping patterns, which have ecological and agronomic significance. Additionally, reliance on the completeness of IACS data means that unmatched fields - about 3 % due to data gaps or ineligible fields - are excluded, possibly underrepresenting certain land-use changes. While IACS data undergoes verification, potential inaccuracies or biases may still exist, particularly due to its focus on subsidized land. However, subsidized farm land had a share of 96.6 % of agricultural land in 2023, showing that our dataset covers the vast majority of relevant land [14]. This could affect representativeness and may introduce uncertainties in analyses reliant on this dataset.

Third, the method introduces inaccuracies in total crop shares for all years except the base year. As shown in Fig. 1, field geometries often split differently across years, and only the crop in the largest overlapping split is considered. Consequently, summing crop shares of individual years may not match sums from the same years after applying the largest overlap approach.

Acknowledging these limitations is crucial for the accurate interpretation and application of the crop sequence dataset (Table 1, Table 2, Table 3).

**Table 1:** Provides an overview of the data structure and field descriptions.

Column name	Data type	Description	Example value
geometry	Polygon	Field boundary geometry in WGS84	POLYGON((...))
ID	Integer	Unique identifier for each field	12,345
AREA_HA	Float	Area of the field in hectares	2.45
CODE_2019	Integer	Crop type code for 2019	131
CODE_2020	Integer	Crop type code for 2020	115
CODE_2021	Integer	Crop type code for 2021	411
CODE_2022	Integer	Crop type code for 2022	131
CODE_2023	Integer	Crop type code for 2023	115
CODE_2024	Integer	Crop type code for 2024	411

**Table 2:** Contents of the crop sequence dataset. The first 5 rows are shown.

ID	Geometry	AREA_HA	CODE_2019	CODE_2020	CODE_2021	CODE_2022	CODE_2023	CODE_2024
1	POLYGON ((...	1.5183	411.0	115.0	411.0	115.0	311.0	411
2	POLYGON ((...	0.0737	411.0	115.0	411.0	115.0	915.0	915
3	POLYGON ((...	5.8167	459.0	459.0	459.0	459.0	459.0	459
4	POLYGON ((...	1.3957	459.0	459.0	459.0	459.0	459.0	459
5	POLYGON ((...	1.0079	411.0	115.0	411.0	115.0	311.0	411

## Ethics Statement

The authors have read and followed the ethical requirements for publication in Data in Brief and confirm that the current work does not involve human subjects, animal experiments, or any data collected from social media platforms.

## CRediT Author Statement

**Christoph Pahmeyer:** Conceptualization, Supervision, Methodology Chapter, Software Chapter, Writing-Chapter, Writing-Reviewing and Editing, **Till Kuhn:** Conceptualization, Supervision, Writing-Original draft preparation, Writing-Reviewing and Editing, **Hugo Storm:** Methodology Chapter, Software Chapter, Writing-Reviewing and Editing.

## Declaration of Generative AI and AI-assisted Technologies in the Writing Process

During the preparation of this work the authors used both Anthropic’s Claude and Microsoft's Copilot assistant in order to improve the readability of the manuscript. After using this service, the authors reviewed and edited the content as needed and take full responsibility for the content of the published article.

## Data Availability

ZenodoA crop sequence dataset of the German federal state of North Rhine-Westphalia from 2019-2024 (Original data).
